# Bayesian risk prediction model for colorectal cancer mortality through integration of clinicopathologic and genomic data

**DOI:** 10.1038/s41698-023-00406-8

**Published:** 2023-06-10

**Authors:** Melissa Zhao, Mai Chan Lau, Koichiro Haruki, Juha P. Väyrynen, Carino Gurjao, Sara A. Väyrynen, Andressa Dias Costa, Jennifer Borowsky, Kenji Fujiyoshi, Kota Arima, Tsuyoshi Hamada, Jochen K. Lennerz, Charles S. Fuchs, Reiko Nishihara, Andrew T. Chan, Kimmie Ng, Xuehong Zhang, Jeffrey A. Meyerhardt, Mingyang Song, Molin Wang, Marios Giannakis, Jonathan A. Nowak, Kun-Hsing Yu, Tomotaka Ugai, Shuji Ogino

**Affiliations:** 1 Program in MPE Molecular Pathological Epidemiology, Department of Pathology, Brigham and Women’s Hospital and Harvard Medical School, Boston, MA USA; 2 Department of Medical Oncology, Dana-Farber Cancer Institute and Harvard Medical School, Boston, MA USA; 3 Cancer and Translational Medicine Research Unit, Medical Research Center Oulu, Oulu University Hospital, and University of Oulu, Oulu, Finland; 4 Broad Institute of MIT and Harvard, Cambridge, MA USA; 5 Department of Pathology, Center for Integrated Diagnostics, Massachusetts General Hospital and Harvard Medical School, Boston, MA USA; 6 Genentech/Roche, South San Francisco, CA USA; 7 Department of Epidemiology, Harvard T.H. Chan School of Public Health, Boston, MA USA; 8 Department of Nutrition, Harvard T.H. Chan School of Public Health, Boston, MA USA; 9 Clinical and Translational Epidemiology Unit, Massachusetts General Hospital and Harvard Medical School, Boston, MA USA; 10 Division of Gastroenterology, Massachusetts General Hospital, Boston, MA USA; 11 Channing Division of Network Medicine, Department of Medicine, Brigham and Women’s Hospital and Harvard Medical School, Boston, MA USA; 12 Department of Immunology and Infectious Diseases, Harvard T.H. Chan School of Public Health, Boston, MA USA; 13 Department of Biostatistics, Harvard T.H. Chan School of Public Health, Boston, MA USA; 14 Department of Medicine, Brigham and Women’s Hospital and Harvard Medical School, Boston, MA USA; 15 Department of Biomedical Informatics, Harvard Medical School, Boston, MA USA; 16 Cancer Immunology and Cancer Epidemiology Programs, Dana-Farber Harvard Cancer Center, Boston, MA USA

**Keywords:** Computational biology and bioinformatics, Cancer genomics, Prognostic markers, Colorectal cancer

## Abstract

Routine tumor-node-metastasis (TNM) staging of colorectal cancer is imperfect in predicting survival due to tumor pathobiological heterogeneity and imprecise assessment of tumor spread. We leveraged Bayesian additive regression trees (BART), a statistical learning technique, to comprehensively analyze patient-specific tumor characteristics for the improvement of prognostic prediction. Of 75 clinicopathologic, immune, microbial, and genomic variables in 815 stage II–III patients within two U.S.-wide prospective cohort studies, the BART risk model identified seven stable survival predictors. Risk stratifications (low risk, intermediate risk, and high risk) based on model-predicted survival were statistically significant (hazard ratios 0.19–0.45, vs. higher risk; *P* < 0.0001) and could be externally validated using The Cancer Genome Atlas (TCGA) data (*P* = 0.0004). BART demonstrated model flexibility, interpretability, and comparable or superior performance to other machine-learning models. Integrated bioinformatic analyses using BART with tumor-specific factors can robustly stratify colorectal cancer patients into prognostic groups and be readily applied to clinical oncology practice.

## Introduction

Colorectal cancer develops in the context of a complex interplay between the host, microbes, and neoplastic cells in the local intestinal microenvironment^1^. Survival prediction based solely on tumor-node-metastasis (TNM) staging is imperfect due to tumor heterogeneity as well as inaccurate assessment of tumor spread. Within stage II/III patients, risk assessment has crucial implications on the use of adjuvant chemotherapy, as well as treatment intensity and duration^2,3^. Hence, large-scale multivariable analyses of factors that contribute to tumor progression are necessary to better predict outcomes of individual patients. Accumulating evidence indicates that factors such as tumor microsatellite instability (MSI) status, *BRAF* mutation, the amount of *Fusobacterium nucleatum*, and T-cell infiltrates are relevant prognostic biomarkers in colorectal cancer^4–6^. Considering these findings, we hypothesized that the integration of tumor and immune characteristics with TNM classification could improve a prognostic prediction model in colorectal cancer.

To utilize available clinicopathological variables in survival prediction, we implemented an ensemble sum-of-trees classification model, Bayesian additive regression trees (BART). Ensemble methods enable flexible modeling of nonlinear and interactive relationships between predictors and outcome variables while maintaining model interpretability through variable importance measures^7^, and have yielded promising results in tumor molecular subtype classification, therapy response, and survival prediction across multiple cancer types^8–10^. BART extends the classical ensemble tree paradigm by introducing an underlying probabilistic distribution to a sum-of-trees model, allowing for inherent regularization. BART has demonstrated favorable performance and superior variable selection capabilities compared to other machine-learning methods, including random forest (RF), gradient boosting (GB), least absolute shrinkage and selection operator (LASSO), multivariate adaptive regression spline, and artificial neural networks (ANN)^11^, and has delivered promising results in prior studies in proteomic profiling, gene regulatory network analysis, and nonparametric survival analysis^12–14^.

In this study, we constructed a BART model that incorporated TNM stage components with other factors to improve mortality risk stratification in stage II/III patients, utilizing a colorectal cancer patient database in two large prospective cohort studies, namely the Nurses’ Health Study (NHS) and the Health Professionals Follow-up Study (HPFS). We confirmed good BART model performance, indicated by the receiver operating characteristics (ROC) curve in comparison to RF, GB, and other statistical learning methods, and externally validated by using The Tumor Genome Atlas (TCGA) dataset. We examined variables that contribute to the BART models in terms of stability of significance by permutation test across fivefold cross-validation, as well as partial dependency of outcome on important variables. Our study has demonstrated that Bayesian ensemble models can integrate a variety of tumor and patient-specific factors to improve survival prediction and can serve as clinical tools to assess individual’s risk for cancer mortality, thereby adding precision to optimal patient management.

## Results

### BART model stability

**Fig. 1:**
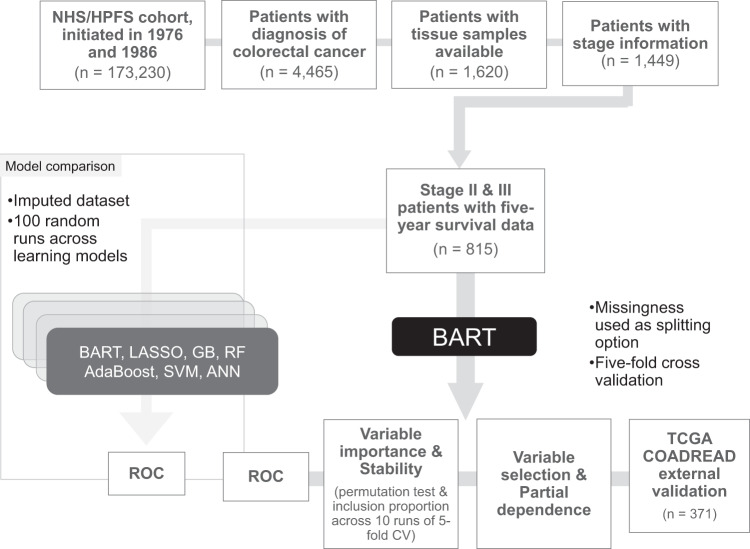
Overview of study. External validation of the BART model was conducted using 106 of 371 stage II–III patients in TCGA dataset as 5-year overall survival information was missing in 265 patients. Overall survival analyses were conducted using all 371 patients with predicted probabilities of 5-year survival status based on the covariates. AdaBoost adaptive boosting, ANN artificial neural network, BART Bayesian additive regression trees, COADREAD colorectal adenocarcinoma, CV cross-validation, GB gradient boosting, HPFS Health Professionals Follow-up Study, LASSO least absolute shrinkage and selection operator, NHS Nurses’ Health Study, RF random forest, ROC receiver operating characteristics, SVM support vector machine, TCGA The Cancer Genome Atlas.

**Fig. 2:**
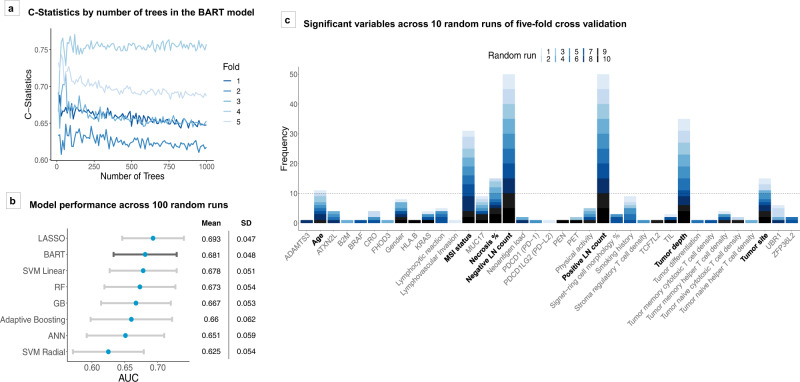
BART model characteristics and performance metrics. **a** Model performances in terms of receiver operating characteristics (ROC) C-statistics for stage II–III 5-year survival models across fivefold cross-validation, with variable number of trees parameter. **b** Model performances across 100 random runs in terms of area under the ROC curve (AUC). Blue dots represent mean AUC values across the runs by model type. Gray bars represent the standard deviations of AUC values across runs. **c** Variable selection using BART at threshold of *P* = 0.05. Figure shows number of times variables were deemed significant across ten random runs. Variables that appeared an average of at least once per fivefold cross-validation were used for downstream analysis. ANN artificial neural network, AUC area under the ROC curve, BART Bayesian additive regression trees, CRO Crohn’s-like reaction, GB gradient boosting, LASSO least absolute shrinkage and selection operator, LNs lymph nodes, MSI microsatellite instability, PEN periglandular reaction, PET peritumoral reaction, RF random forest, ROC receiver operating characteristics, SD standard deviation, SVM support vector machine, TIL tumor-infiltrating lymphocytes.

To construct a Bayesian additive regression trees (BART) model for mortality risk prediction, we included 815 patients with stage II–III colorectal adenocarcinoma derived from a database in the Nurses’ Health Study (NHS) and the Health Professionals Follow-up Study (HPFS) (Fig. 1). Table 1 summarizes patient characteristics. A test of BART model stability by the number of trees set across fivefold cross-validation demonstrated that BART reached performance stability prior to 500 trees (Fig. 2a). Thus, 500 was set as the default number of trees for the remainder of the study to ensure stability and consistency across models.

**Table 1:** Patient characteristics.

		TNM stage		
Characteristic^a^	All cases (*N* = 815)	Stage II (*N* = 453)	Stage III (*N* = 362)	*P* value^b^
Sex				0.036
Female (NHS)	502 (62%)	294 (65%)	208 (57%)	
Male (HPFS)	313 (38%)	159 (35%)	154 (43%)	
Mean age ± SD (years)	68.6 ± 8.9	68.9 ± 8.7	68.1 ± 9.1	0.22
Family history of colorectal cancer in any first-degree relative				0.73
No	660 (81%)	365 (81%)	295 (82%)	
Yes	152 (19%)	87 (19%)	65 (18%)	
Pack-years of smoking at diagnosis				0.24
0	328 (42%)	174 (40%)	154 (44%)	
1–19	178 (23%)	94 (22%)	84 (24%)	
20–39	137 (18%)	80 (18%)	57 (16%)	
≥40	137 (18%)	85 (20%)	52 (15%)	
Tumor location				0.23
Cecum	136 (17%)	76 (17%)	60 (17%)	
Ascending to transverse colon	288 (36%)	173 (38%)	115 (32%)	
Descending to sigmoid colon	277 (34%)	146 (32%)	131 (36%)	
Rectum	108 (13%)	55 (12%)	53 (15%)	
Tumor differentiation				0.077
Well to moderate	709 (87%)	403 (89%)	306 (85%)	
Poor	106 (13%)	50 (11%)	56 (15%)	
Tumor depth of invasion (pT stage)				<0.0001
pT1	13 (2%)	0 (0%)	13 (4%)	
pT2	45 (5%)	0 (0 %)	47 (13%)	
pT3	704 (87%)	423 (93%)	281 (78%)	
pT4	49 (6%)	30 (7%)	19 (5%)	
Positive lymph node count				<0.0001
0 (pN0)	416 (56%)	416 (100%)	0 (0%)	
1–3 (pN1)	228 (30%)	0 (0%)	228 (69%)	
≥4 (pN2)	103 (14%)	0 (0%)	103 (31%)	
Negative lymph node count				<0.0001
0–5	144 (21%)	55 (15%)	89 (28%)	
6–11	243 (35%)	125 (34%)	118 (37%)	
12–17	137 (20%)	77 (21%)	60 (19%)	
≥18	170 (24%)	114 (31%)	56 (17%)	
Extent of extraglandular necrosis				0.29
0%	509 (62%)	285 (63%)	224 (62%)	
1–19%	167 (20%)	85 (19%)	82 (23%)	
≥20%	139 (17%)	83 (18%)	56 (15%)	
Lymphovascular invasion				0.003
None	538 (90%)	320 (94%)	218 (86%)	
Mild	35 (6%)	13 (4%)	22 (9%)	
Moderate/extensive	21 (4%)	7 (2%)	14 (6%)	
Perineural invasion				0.009
None	575 (98%)	333 (99%)	242 (96%)	
Mild	8 (1%)	3 (0.9%)	5 (2%)	
Moderate/extensive	5 (0.9%)	0 (0%)	5 (2%)	
Extracellular mucinous component				0.004
0%	448 (57%)	232 (53%)	216 (62%)	
1–49%	193 (25%)	107 (25%)	86 (25%)	
≥50%	143 (18%)	97 (22%)	46 (13%)	
Signet ring cell component				0.24
0%	677 (87%)	383 (88%)	294 (84%)	
1–9%	75 (10%)	35 (8%)	40 (11%)	
≥10%	29 (4%)	15 (3%)	14 (4%)	
Tumor-infiltrating lymphocytes (TILs)				0.003
Absent/minimal	558 (72%)	294 (68%)	264 (76%)	
Mild	123 (16%)	69 (16%)	54 (16%)	
Moderate/severe	96 (12%)	68 (16%)	28 (8%)	
MSI status				<0.0001
Non-MSI-high	556 (80%)	279 (73%)	277 (88%)	
MSI-high	143 (20%)	104 (27%)	39 (12%)	
CIMP status				0.0004
Low/negative	520 (75%)	268 (70%)	252 (82%)	
High	174 (25%)	117 (30%)	57 (18%)	
Mean LINE-1 methylation level ± SD (%)	63.7 ± 10.2	64.3 ± 10.0	62.9 ± 10.3	0.069
*KRAS* mutation				0.10
Wild-type	390 (58%)	224 (61%)	166 (55%)	
Mutant	278 (42%)	141 (39%)	137 (45%)	
*BRAF* mutation				0.15
Wild-type	584 (83%)	314 (81%)	270 (85%)	
Mutant	122 (17%)	75 (19%)	47 (15%)	
*PIK3CA* mutation				0.38
Wild-type	550 (84%)	293 (83%)	257 (85%)	
Mutant	106 (16%)	62 (17%)	44 (15%)	
Memory cytotoxic T-cell (CD3^+^CD8^+^CD45RO^+^ cell) density^c^				0.014
Q0 (0, lowest)	244 (48%)	115 (42%)	139 (56%)	
Q1	88 (17%)	56 (20%)	32 (14%)	
Q2	88 (17%)	53 (19%)	35 (15%)	
Q3 (highest)	88 (17%)	53 (19%)	35 (15%)	
Memory helper T-cell (CD3^+^CD4^+^CD45RO^+^ cell) density^c^				0.51
Q0 (0, lowest)	176 (34%)	89 (32%)	87 (37%)	
Q1	111 (22%)	64 (23%)	47 (20%)	
Q2	111 (22%)	65 (23%)	46 (20%)	
Q3 (highest)	110 (22%)	59 (21%)	51 (22%)	
*Fusobacterium nucleatum* in tumor				0.34
Negative	572 (85%)	309 (83%)	263 (86%)	
Positive	104 (15%)	62 (17%)	42 (14%)	
*Bifidobacterium* species in tumor				0.46
Negative	485 (70%)	269 (71%)	216 (68%)	
Positive	211 (30%)	110 (29%)	101 (32%)	
Five-year colorectal cancer-specific survival status				<0.0001
Survival	661 (81%)	392 (87%)	269 (74%)	
Death	154 (19%)	61 (13%)	93 (26%)	

*AJCC* American Joint Committee on Cancer, *CIMP* CpG island methylator phenotype, *HPFS* Health Professionals Follow-up Study, *LINE-1* long-interspersed nucleotide element-1, *MSI* microsatellite instability, *NHS* Nurses’ Health Study, *SD* standard deviation, *TNM* tumor-node-metastasis.

^a^Percentage indicates the proportion of patients with a specific clinical, pathologic, or molecular characteristic among all patients or in strata of the stage, excluding missing data.

^b^To compare categorical data between stages, the Chi-square test was performed. To compare continuous variables, analysis of variance (ANOVA) was performed for variables exhibiting normality. For variables that did not follow a normal distribution, values were separated into ordinal categories prior to the Chi-square test. For lymphovascular invasion and perineural invasion, Fisher’s exact tests were performed.

^c^Tumor tissue CD3^+^CD8^+^CD45RO^+^ and CD3^+^CD4^+^CD45RO^+^ cell density measures (cells/mm^2^) were categorized into 0 value (the lowest category, Q0) and positive values divided equally into tertiles (Q1 to Q3).

Clinical, pathologic, molecular, and immune characteristics of patients with colorectal cancer according to AJCC TNM staging.

### Comparison across machine-learning models

A comparison of the BART model to other machine-learning algorithms using multiple random validation on a dataset with imputation of missing values yielded BART as a competitive model across the majority of 100 random runs. BART performance was amongst the top two of eight tested models in terms of mean AUC (area under ROC curve) across runs [mean AUC 0.681, standard deviation (SD) 0.048], following LASSO regression (mean AUC 0.693, SD 0.047) (Fig. 2b). Amongst ensemble models, BART demonstrated the best performance, followed by random forest (mean AUC 0.673, SD 0.054).

### Important variables for survival prediction for stage II–III colorectal cancer

BART stage II–III survival prediction model revealed several statistically significant variables by permutation test at *P* value threshold of 0.05, which was used in this selection procedure (Fig. 2c). Out of the 75 examined variables, 7 variables passed the significance threshold on average at least once within a fivefold cross-validation across 10 random runs (i.e., ≥10 of 50 runs). The most frequently observed were, in descending order, positive lymph node count, negative lymph node count, the depth of tumor invasion (pT stage), MSI status, tumor site, the extent of extraglandular necrosis, and age.

**Fig. 3:**
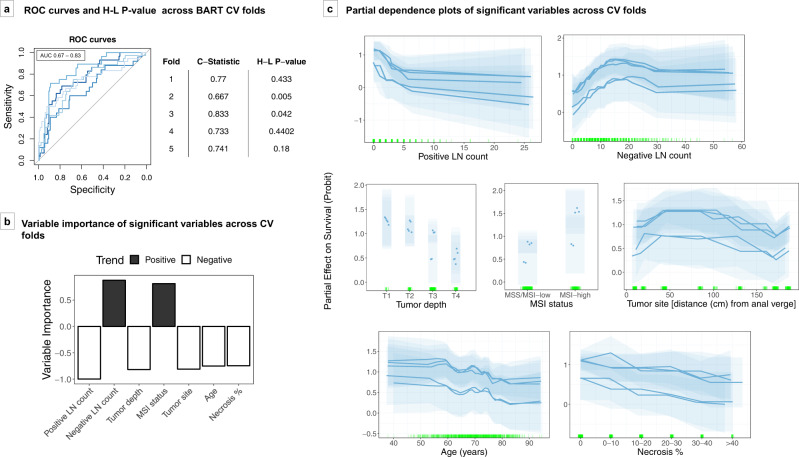
BART stage II–III survival prediction model. The BART prediction model was constructed based on seven significant and stable variables, namely positive and negative lymph node counts, depth of tumor invasion, microsatellite instability (MSI) status, tumor site, extraglandular necrosis, and age. **a** ROC curves and Hosmer–Lemeshow *P* values across fivefolds of cross-validation (CV). **b** Average variable importance across fivefolds of cross-validation, displayed in order of highest average importance. Black bars represent variables with positive trend with survival and white bars represent variables with negative trend with survival. **c** Partial dependence plots of significant variables across cross-validation folds. Each transparent block represents the 95% credible interval of one cross-validation fold based on 1000 posterior samples. Partial effects are plotted in terms of probability of survival on Probit scale. Darker lines and points represent the expected value of partial dependence for each variable across 1000 posterior samples. Green vertical hash marks on the *X* axis indicate observed data points used to generate the model. AUC area under the ROC curve, BART Bayesian additive regression trees, CV cross-validation, H-L Hosmer–Lemeshow, LNs lymph nodes, MSI microsatellite instability, MSS microsatellite stable, ROC receiver operating characteristics.

BART model using these seven significant and stable variables achieved AUCs of 0.67–0.83 (median 0.74) across fivefolds of cross-validation (Fig. 3a). The majority of folds (3/5) demonstrated goodness-of-fit by Hosmer–Lemeshow test. Partial dependence plots of these variables showed that negative lymph node count and MSI status were positively associated with 5-year colorectal cancer-specific survival, whereas positive lymph node count, pT stage, age, extraglandular necrosis, and more proximal tumor site (estimated distance from anal verge) were negatively associated with survival (Fig. 3b, c).

BART model using overall stage, pT stage, or pN stage alone as a predictor achieved median AUCs of 0.47–0.62 across fivefolds of cross-validation, consistently lower than median AUC of 0.74 from BART model using seven significant variables (Supplementary Table 2).

### Risk prediction model demonstrates risk stratification within stage II–III colorectal cancer

**Fig. 4:**
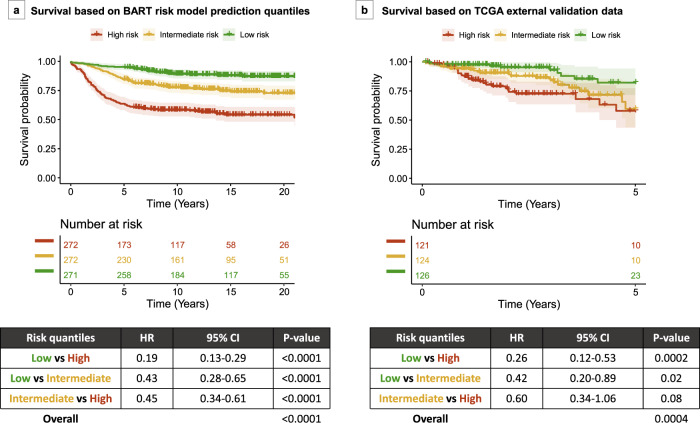
Kaplan–Meier plots for survival in patients with stage II/III colorectal cancer, based on risk quantiles from BART risk model. **a** NHS/HPFS dataset survival based on risk quantiles. **b** TCGA external validation dataset survival based on risk quantiles. Tables show Cox proportional hazards models using risk quantiles and overall *P* values by log-rank test. BART Bayesian additive regression trees, CI confidence interval, HR hazard ratio.

Using BART leave-one-out analysis, as detailed in Methods, stage II–III colorectal cancer patients were separated into three risk quantiles based on predicted probabilities of 5-year survival (low risk if ≥0.884, intermediate risk if ≥0.758 and <0.884, high risk if <0.758). Survival analysis using Cox proportional hazards regression model demonstrated significant survival differences between the risk tertile categories, i.e., low risk vs high risk [hazard ratio (HR) 0.19, 95% confidence interval (CI) 0.13–0.29, *P* value < 0.0001], low risk vs intermediate risk (HR 0.43, 95% CI 0.28–0.65, *P* value < 0.0001), and intermediate risk vs high risk (HR 0.45, 95% CI 0.34–0.61, *P* value < 0.0001), with overall log-rank test *P* value of <0.0001 (Fig. 4a). Risk groups remained significant in a multivariate Cox proportional hazards model adjusting for stage (*P* value < 0.0001, Table 2) as well as a multivariate Cox proportional hazards model adjusting for all independent predictors included in the model (*P* value 0.0008, Table 3).

**Table 2:** Multivariate Cox proportional hazards regression model for risk group and TNM stage.

Cox proportional hazards model	NHS/HPFS (primary dataset)		TCGA (external validation dataset)	
	HR (95% CI)	*P* value	HR (95% CI)	*P* value
Risk group^a^	2.17 (1.79–2.63)	<0.0001	1.64 (1.16–2.33)	0.005
TNM stage				
Stage II	Referent		Referent	
Stage III	1.19 (0.89–1.58)	0.24	1.96 (1.12–3.43)	0.02

*CI* confidence interval, *HPFS* Health Professionals Follow-up Study, *HR* hazard ratio, *NHS* Nurses’ Health Study, *TCGA* The Cancer Genome Atlas, *TNM* tumor-node-metastasis.

^a^Risk group coded as ordinal variable in order of low risk (1), intermediate risk (2), and high risk (3). HR for risk group represents HR for one unit increase in risk group.

Models were constructed based on NHS/HPFS data and TCGA data.

**Table 3:** Multivariate Cox proportional hazards regression model for risk group and independent predictors included in the BART risk model.

Cox proportional hazards model	NHS/HPFS (primary dataset)		TCGA (external validation dataset)	
	HR (95% CI)	*P* value	HR (95% CI)	*P* value
Risk group^a^	1.57 (1.21–2.05)	0.0008	1.77 (1.04–3.00)	0.03
Number of positive lymph nodes	1.11 (1.06–1.16)	<0.0001	0.98 (0.81–1.19)	0.83
Number of negative lymph nodes	1.00 (0.98–1.02)	0.70	0.70 (0.52–0.94)	0.02
Tumor depth	1.33 (0.93–1.89)	0.11	1.74 (1.24–2.43)	0.001
Extraglandular necrosis	1.02 (0.90–1.16)	0.73	1.00 (0.97–1.03)	0.94
Age at diagnosis	1.02 (1.00–1.04)	0.03	1.40 (1.10–1.77)	0.006
MSI status				
Non-MSI-high	Referent		Referent	
MSI-high	0.36 (0.18–0.75)	0.006	1.02 (0.33–3.18)	0.98
Tumor site	1.00 (1.00–1.00)	0.80	1.05 (0.80–1.40)	0.72

*BART* Bayesian additive regression trees, *CI* confidence interval, *HPFS* Health Professionals Follow-up Study, *HR* hazard ratio, *MSI* microsatellite instability, *NHS* Nurses’ Health Study, *TCGA* The Cancer Genome Atlas, *TNM* tumor-node-metastasis.

Models were constructed based on NHS/HPFS data and TCGA data.

^a^Risk group coded as ordinal variable in order of low risk (1), intermediate risk (2), and high risk (3). HR for risk group represents HR for one unit increment in risk group.

HRs for number of positive lymph nodes and number of negative lymph nodes represent HRs for 1 node increment. HR for tumor depth represents HR for 1 pT stage increment. HR for extraglandular necrosis represents one unit increment in ordinal category of extraglandular necrosis (0%, <10%, <20%, <30%, <40%, and ≥40%). HR for age at diagnosis represents HR for 1 year increment. HR for tumor site represents HR for 1 cm increment in distance from the anal verge based on the colorectal continuum model.

**Fig. 5:**
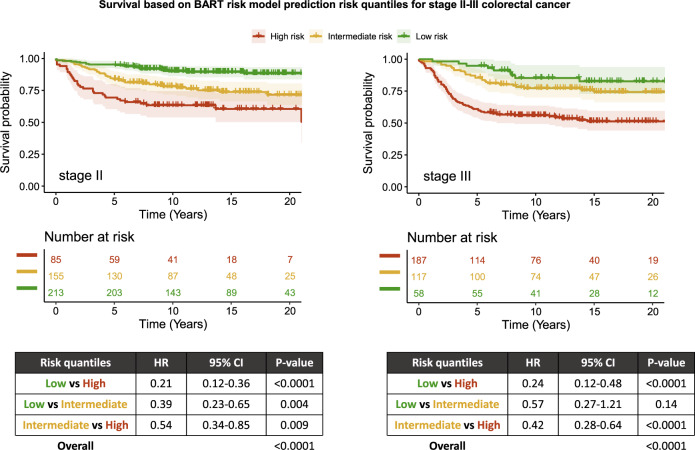
Stage-specific Kaplan–Meier plots for survival. Survival plots are shown for patients with stage II (left) and stage III (right) colorectal cancer, based on risk quantiles derived from predicted probabilities generated by the BART risk model. Table shows Cox proportional hazards model using risk quantiles and overall *P* value by log-rank test. BART Bayesian additive regression trees, CI confidence interval, HR hazard ratio.

Exploratory analyses using stratification by both risk quantiles and stage demonstrated decreasing HR compared to high-risk stage III (reference) in the following order: high-risk stage II (*P* value 0.26), intermediate-risk stage III, intermediate-risk stage II, low-risk stage III, and low-risk stage II (*P* values < 0.0001) (Supplementary Fig. 1). Stage-specific analyses demonstrated that mortality risk differences were significant for low risk vs high risk and low risk vs intermediate risk in stage II patients and for low risk vs high risk and intermediate risk vs high risk in stage III patients (*P* values < 0.005), and suggestive for intermediate risk vs high risk in stage II patients (*P* values between 0.005 and 0.05) (Fig. 5).

### External validation with TCGA

An external validation with TCGA data showed that the BART risk prediction model achieved an AUC of 0.68 based on 106 of 371 stage II–III patients with 5-year overall survival information (i.e., patients who died within 5 years or survived for at least 5 years) (Supplementary Fig. 2). Five-year overall survival was used as a surrogate endpoint and censoring was set at 5 years (see “Methods”) as colorectal cancer-specific survival information was not available. The full TCGA dataset of 371 stage II–III colorectal cancer patients was separated into three risk quantiles based on predicted probabilities of 5-year survival status (low risk if ≥0.662, intermediate risk if ≥0.517 and <0.662, high risk if <0.517) and incorporated into a Cox proportional hazards model. The model yielded a significant difference between low-risk and high-risk quantiles (HR 0.26, 95% CI 0.12–0.53, *P* value 0.0002) and suggestive evidence of the difference between low-risk and intermediate-risk quantiles (HR 0.42, 95% CI 0.20–0.89, *P* value 0.02), with a log-rank test *P* value of 0.0004 across the quantiles (Fig. 4b). Risk groups remained significant at level of suggestive evidence in a multivariate Cox proportional hazards model adjusting for stage (*P* value 0.005, Table 2) as well as a multivariate Cox proportional hazards model adjusting for all independent predictors included in the model (*P* value 0.03, Table 3).

**Fig. 6:**
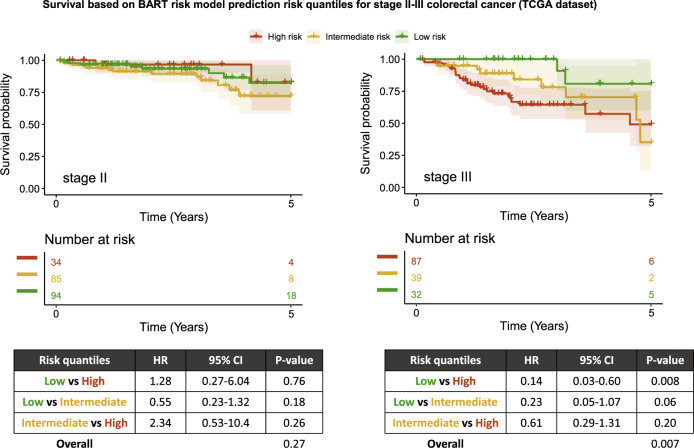
Stage-specific Kaplan–Meier plots for survival in TCGA dataset. Survival plots are shown for patients with stage II (left) and stage III (right) colorectal cancer in TCGA dataset, based on risk quantiles derived from predicted probabilities generated by the BART risk model. Table shows Cox proportional hazards model using risk quantiles and overall *P* value by log-rank test. BART Bayesian additive regression trees, CI confidence interval, HR hazard ratio.

Separate analysis based on stage II or stage III only data demonstrated that 5-year overall survival suggestively differed between low-risk and high-risk groups in stage III patients (*P* value 0.008); however, they did not demonstrate any level of significance for stage II patients (Fig. 6).

### Experimental risk prediction calculator based on BART risk model

A risk prediction calculator interface is shown in Supplementary Fig. 3, which takes as input the seven significant and stable variables, allows for missing values, and outputs the survival probability and risk group (low risk, intermediate risk, or high risk) for each patient in question. An experimental version of the BART risk prediction model is available for download at https://github.com/mm-zhao/BART.

## Discussion

In this multivariable study on the colorectal cancer survival prediction, BART demonstrated comparable model performance across multiple random runs compared to other nonlinear learning models and LASSO linear regression. Within BART models, the most stable predictors for 5-year colorectal cancer-specific survival in stage II–III were positive lymph node count, negative node count, depth tumor of invasion, MSI status, tumor site, age, and extent of extraglandular necrosis. All variables can be available in routine clinical assessment of colorectal cancer if a pathologist (or artificial intelligence algorithm/digital image analysis) can record the extent of extraglandular necrosis, which is the least contributor among the seven variables. A risk prediction model based on these variables was constructed to categorize patients into low-, intermediate-, and high-risk groups.

Rapid developments in colorectal cancer research have prompted the inclusion of molecular factors, such as MSI status and mutations in *KRAS* and *BRAF*, as important features for guiding cancer treatment in stage II–IV patients in the most recent edition of the AJCC (American Joint Committee on Cancer) Cancer Staging Manual^15^. While staging in colorectal cancer is currently based entirely on anatomical features, alternative classification schemes, such as the Immunoscore, have demonstrated good utility in classifying patient prognosis based on T-cell density quantiles^16^. Within stage II and stage III colorectal cancer, where classification has strong implications on treatment strategies, staging is a pivotal yet challenging matter. Thus, the addition of prognostic factors beyond anatomical tumor spread in a standardized risk model can help refine diagnosis and offer additional patient-specific survival information for clinical management.

Applications of statistical learning algorithms in cancer classification and prognosis prediction have gained traction in the recent decade due to their ability to model complex relationships in a high-dimensional context. In recent years, ANN-based algorithms have gained momentum in cancer research, particularly in image-based studies, according to a literature survey by Kourou et al.^17^. Compared to ANN-based models, ensemble classification and regression trees, though less prevalent in the cancer literature, have particular advantages as flexible learning models that require few tuning parameters and allow for variable-level model interpretations. These algorithms have demonstrated superior performance in handling heterogeneous datasets compared to deep learning methods^17^, with overall better performance in a systematic review across learning models^18^. We tested the performance of BART against a range of learning models in our study dataset. We found that ensemble methods were more favorable in ROC performance compared to SVM and ANN with a single hidden layer, and that BART was the preferable ensemble method across 100 random runs. LASSO linear regression performed marginally better than BART across runs in our dataset; however, BART is overall a more flexible and adaptable model in comparison, as LASSO models require a priori manual addition of interactions and lack the ability to model nonlinear relationships or handle missing values.

Ensemble methods maintain model interpretability through variable importance and partial dependence measures. An extension upon variable importance measures using permutation test, a form of which was used in this study, has demonstrated a reduction of variable selection bias and robustness in analyses of high-dimensional datasets^19^. We found that BART can be used to identify influential variables for predictions of colorectal cancer stage classification and colorectal cancer-specific survival in a robust manner. Many of the chosen variables are known to be important prognostic factors in literature, demonstrating that BART can reliably select meaningful variables for prediction of survival. From a set of the 75 candidate features, including clinical, epidemiological, immunologic, microbial, and tumor molecular factors, the BART model robustly isolated a subset of contributing variables over fivefold cross-validation and random runs. Using posterior sampling based on BART’s Bayesian probabilistic model, we were able to estimate credible intervals of individual variable influence on outcome, as illustrated through partial dependence plots. Thus, we could capture both the trend of variable influence and the level of certainty associated with influence within the models.

Our analyses showed that the intermediate-risk group was statistically significant in survival compared to low and high risk groups in the primary dataset. However, this significance is not as robustly reflected in external validation with TCGA data, particularly within substage analyses. The external validation and substage analyses may be underpowered, though the trend is suggestive and consistent with the primary data. The intermediate-risk category may warrant a more aggressive level of clinical management than those from the low-risk category, though this remains to be further studied in terms of treatment implications in the clinical setting.

Partial dependence plots of important variables in the BART models demonstrated relationships between predictor variables and outcome consistent with those previously reported in the literature, including MSI status and negative lymph node count as favorable prognosticators and extraglandular necrosis as an unfavorable prognosticator in colorectal cancer^20–22^. Furthermore, the partial dependence plots highlight the nonlinear nature of relationships between several variables and survival, such as worse survival for tumors arising from the ascending colon compared to other sites.

Within stage II, where high-risk factors and staging strongly influence clinical decision for chemotherapy^23^, our results confirm that variables apart from those traditionally used in TNM staging can be used in the clinical setting to help predict and refine prognosis. Several guidelines issued by the National Comprehensive Cancer Network (NCCN) suggest that stage II tumors with high-risk features, such as lymphovascular invasion, perineural invasion, less than 12 lymph nodes examined, positive surgical margins, and poor tumor differentiation, could benefit from adjuvant chemotherapy^24^. However, there currently exists no clinical standard for the identification of high-risk stage II colorectal cancer, an issue compounded by the multitude of variables and their interrelationships that can influence survival in colorectal cancer. A study by Babcock et al. noted that not all high-risk features have the same adverse effects on colorectal cancer survival, with pT4 tumors in combination with other high-risk features denoting the most survival benefit from adjuvant chemotherapy^25^. Through variable inclusion proportions and partial dependence plots in the BART models, we found that selected features have variable degrees of impact on patient survival. For instance, variables such as positive lymph node count, negative node count, and depth of tumor invasion have more stable and robust influences on survival than tumor site. Nonetheless, a larger dataset is clearly needed to better evaluate the prognostic role of detailed tumor location and modifying effect of tumor pathological features^26^, which may further contribute to a prognostic stratification of patients in the future. A predictive model with intrinsic weighing of key variables may thus be used to help standardize risk assessment, functioning as a risk calculator to guide clinical decisions, akin to other established models for risk prediction in colorectal cancer^27,28^. It remains to be determined how various treatment modalities can be incorporated into robust mortality risk prediction models.

In recent years, the use of statistical learning models to stratify patient risk based pathology slide-level data through deep learning methods or the aggregation of multiple influential factors have demonstrated success in predicting prognosis to a level of precision beyond what was previously achievable using single key variables, such as tumor depth, MSI status, and tumor-infiltrating lymphocyte scoring. For example, an artificial intelligence (AI) based immunoscore was constructed from a deep learning model using hematoxylin and eosin (H&E) and immunohistochemical stains of immune subtypes from patients with all stages of colorectal cancer, and was found in a multivariate Cox proportional hazards model to significantly stratify patients into prognostic groups^29^. Other methods such as using random forest or generalized linear models to aggregate multiple clinical variables and gene expression in colorectal cancer demonstrated AUC of around 0.7–0.8 in predicting survival^30^. While many existing models aggregate patients of all stages, including local (stage I) tumors and metastatic (stage IV) tumors, our BART risk model concentrates on the stage II/III population of patients with colorectal cancer to provide meaningful, fine-tuned risk stratification for patients where treatment with adjuvant chemotherapy currently depends heavily on the presence of lymph node metastasis, which is subjected to sampling error, and treatment intensity and duration depends on risk assessment, which currently lacks standardization^3^. By focusing on this group of patients, we aimed to create a model that has clear and immediate clinical utility in the current treatment landscape for colorectal cancer. Furthermore, the use of slide-based information alone using deep learning models or an ensemble of deep learning models have demonstrated the ability to distinguish high-risk and low-risk groups in stage II/III patients with colorectal cancer^31,32^. Future developments, including the incorporation of deep learning methods to learn specific slide-based features rather than manual grading of slides features, such as extent of extraglandular necrosis, would help preserve model interpretability while further increasing the efficiency and consistency, and thus utility, of the current version of the risk model described in this study.

External validation using The Cancer Genome Atlas (TCGA) dataset demonstrated that our Bayesian risk model may be generalizable to other datasets with conserved utility and the ability to separate patients into statistically significant risk groups. However, with missing information on colorectal cancer-specific survival and shorter follow-up times, TCGA dataset could not be used optimally at this time as a validation set. Another existing dataset, the Surveillance, Epidemiology, and End Results (SEER) program, lacks detailed tumor characteristics information. Ongoing efforts in data collection and incorporation of more clinical, epidemiological, and molecular variables in cancer registries can help provide valuable validation data in future studies.

Other limitations of this study include that, though our study attempted to incorporate several pertinent and established high-risk features for stage II, such as lymphovascular invasion and perineural invasion, the degree of missingness and measurement uncertainty in the collection of these data might have impacted their measurable influence within our models. When more data become available, these variables would be of great interest to examine alongside features found important in this study. Similarly, as we have applied immune density measurements and whole exome sequencing (WES) to a subset of colorectal cancers in our cohort datasets, it may be interesting to incorporate more comprehensive immune and mutational profiles as predictors in future models. Though the BART models in this study focus on colorectal cancer-specific survival to reduce the possible noise and confounders associated with measurements of overall survival, other modifications and considerations can be helpful. For example, as treatment information was not available for this study, we had no means to ascertain the relationship between treatments received based on staging and survival. Thus, we could not determine if survival within stage II might have been affected by the addition of adjuvant therapy. While the extent of extraglandular necrosis was assessable using TCGA H&E slides, the histopathological assessment of each case was generally limited to one slide often with small amounts of tissue. Thus, sampling variability might limit a representation of the degree of necrosis. Studies using multidimensional datasets that include the evaluation of treatment information would help elucidate the relationship between treatment and survival in the context of risk classification within stage II colorectal cancer.

There are notable strengths in our study. First, our molecular pathological epidemiology research database of colorectal cancer patients includes many possible prognosticators, allowing for comprehensive multivariable assessments and comparisons^33,34^. Second, our patient population represents colorectal cancer cases that had occurred in well-established US-wide prospective cohort studies. Accordingly, our subjects included patients who underwent cancer resection and treatment in diverse regions and types of hospitals with little evidence for selection bias^35^, which increases generalizability of findings. Furthermore, we performed comprehensive and rigorous assessments of tested models in terms of prediction performance and interpretability. Through this study, we have illustrated the ability of BART models, by employing Bayesian frameworks within an ensemble sum-of-trees architecture, to provide insight on the degree of certainty and reliably detect the prominent variables contributing to survival from a comprehensive list of potential variables.

In conclusion, statistical learning models that simultaneously integrate multiple variables with consideration for nonlinearity have demonstrated good performance in the prediction of colorectal cancer-specific survival. Ensemble methods such as BART enable model flexibility along with interpretability to identify variables that contribute to patient survival. Focused studies on the identified variables can help elucidate mechanisms of disease progression, and incorporation of these variables into or alongside the current existing staging system can result in a more precise prognostic stratification to guide treatment for patients with colorectal cancer.

## Methods

### Study population

The study was conducted using two ongoing prospective cohort studies in the U.S., the Nurses’ Health Study (NHS), which was initiated in 1976 and enrolled 121,701 registered female nurses aged 30–55 years at baseline, and the Health Professionals Follow-up Study (HPFS), which was initiated in 1986 and enrolled 51,529 male health professionals aged 40–75 at baseline^36^. For both cohorts, questionnaires were sent on a biannual basis to assess demographic, lifestyle, medical, and other pertinent health information. Detailed diet data were collected every 4 years through semiquantitative food frequency questionnaires. The response rate has been more than 90% for each follow-up questionnaire cycle in both cohort studies. Participants had been asked to provide information on diet and lifestyle factors such as height, weight, smoking, use of aspirin and other nonsteroidal anti-inflammatory drugs, alcohol consumption, and red meat consumption. In both studies, the National Death Index was used to ascertain deaths of study participants and identify unreported lethal colorectal cancer cases.

Based on the colorectal continuum model^37^, participants who developed either colon or rectal adenocarcinomas during the study periods were included in this study. Written informed consent was obtained from all study participants. Participating physicians, who were blinded to exposure data, reviewed medical records of identified colorectal cancer cases to confirm the disease diagnosis (i.e., colorectal adenocarcinoma) and to collect data on clinicopathological characteristics including tumor size, tumor anatomical location, AJCC TNM stage, the numbers of lymph nodes positive and negative for tumor metastasis, and cause of death (in deceased patients). Tumor site information (the cecum, ascending colon, hepatic flexure, transverse colon, splenic flexure, descending colon, sigmoid colon, rectosigmoid junction, and rectum) was translated into average distance from the anal verge based on published data on computed tomographic colonography^38,39^. Archival formalin-fixed paraffin-embedded (FFPE) tumor tissue for 1620 participants diagnosed with colorectal adenocarcinoma could be obtained from institutions where tumor resections were performed. We included 815 patients with stage II and III colorectal cancer in our current analysis (Fig. 1). Written informed consent was obtained from all study subjects. The study protocol was approved by the institutional review boards of the Brigham and Women’s Hospital and Harvard T.H. Chan School of Public Health (Boston, MA, USA), and those of participating registries as required.

### Histopathologic analyses

A single pathologist (S.O.), blinded to other data, performed a thorough pathological review of hematoxylin and eosin-stained tissue sections of all colorectal carcinoma cases and recorded the histopathologic features, including tumor differentiation, patterns and degrees of lymphocytic reactions, lymphovascular invasion, perineural invasion, and the extent in percentages (from 0 to 100%) of signet ring cell component, extracellular mucin, and extraglandular necrotic area. All of these features were separately recorded^40^. The proportions were further categorized based on quantiles for signet ring cell percentage and ordinal bins (10% increments) for mucinous percentage (up to 100%, 11 categories) and extraglandular necrotic area (up to 40%, 6 categories). Tumor differentiation was categorized as well to moderate (>50% glandular area) or poor (≤50% glandular area). Four components of histopathological lymphocytic reaction to tumor, tumor-infiltrating lymphocytes (TIL), intratumoral periglandular reaction, peritumoral lymphocytic reaction, and Crohn’s-like lymphoid reaction, were recorded as previously described^41^. Briefly, TIL was defined as lymphocytes on top of tumor cells, intratumoral periglandular reaction was defined as lymphoid reaction in tumor stroma within tumor mass, peritumoral lymphocytic reaction was defined as discrete lymphoid reactions surrounding tumor, and Crohn’s-like reaction was defined as transmural lymphoid reaction. Each of the four lymphocytic reaction components was scored as 0 to 3 (absent/minimal, mild, moderate, and strong), and the overall lymphocytic reaction score (0–12) was the sum of scores for the above four reaction components.

### Tumor molecular analyses

Genomic DNA was extracted from archival FFPE tissue sections of colorectal carcinoma and normal tissue using the QIAamp DNA FFPE Tissue Kit (Qiagen, Hilden, Germany). Tumor MSI status was analyzed using polymerase chain reaction (PCR) of 10 microsatellite markers (D2S123, D5S346, D17S250, BAT25, BAT26, BAT40, D18S55, D18S56, D18S67, and D18S487), and MSI-high was defined as presence of instability in ≥30% of the markers^37^. Methylation statuses of eight CpG island methylator phenotype (CIMP)-specific promoters (*CACNA1G*, *CDKN2A*, *CRABP1*, *IGF2*, *MLH1*, *NEUROG1*, *RUNX3*, and *SOCS1*) and long-interspersed nucleotide element-1 (LINE-1) was determined using bisulfite-treated DNA^37^. CIMP-high was defined as ≥ 5 methylated promoters of eight promoters, and CIMP-low/negative as 0–4 methylated promoters. PCR and pyrosequencing were performed for *KRAS* (codons 12, 13, 61, and 146), *BRAF* (codon 600), and *PIK3CA* (exons 9 and 20)^42^. The PCR primers were 5′-NNNGGCCTGCTGAAAATGACTGAA-3′ (for forward primer) and 5′-[Bio TEG]TTAGCTGTATCGTCAAGGCACTCT-3’ (for reverse primer) for amplifying *KRAS* codons 12 and 13, 5′-biotin-TGGAGAAACCTGTCTCTTGGATAT-3′ (for forward primer) and 5′-TACTGGTCCCTCATTGCACTGTA-3′ (for reverse primer) for amplifying *KRAS* codon 61, 5′-ATGGAATTCCTTTTATTGAAACATC-3′ (for forward primer) and 5′-biotin-TTGCAGAAAACAGATCTGTATTTAT-3′ (for reverse primer) for *KRAS* codon 146, 5′-CAGTAAAAATAGGTGATTTTG-3′ (for forward primer) and 5′-biotin-CAACTGTTCAAACTGATGGG-3′ (for reverse primer) for *BRAF* codon 600, 5′-biotin-AACAGCTCAAAGCAATTTCTACAC-3′ (for forward primer) and 5′-ACCTGTGACTCCATAGAAAATCTT-3′ (for reverse primer) for *PIK3CA* exon 9, and 5′-biotin-CAAGAGGCTTTGGAGTATTTCA-3′ (for forward primer) and 5′-CAATCCATTTTTGTTGTCCA-3′ (for reverse primer) for *PIK3CA* exon 20. The sequencing primers were 5′-TGTGGTAGTTGGAGCTG-3′ (PF1), 5′-TGTGGTAGTTGGAGCT-3′ (PF2), and 5′- TGGTAGTTGGAGCTGGT-3′ (PF3) for *KRAS* codons 12 and 13, 5′-TCATTGCACTGTACTCCTC-3′ for *KRAS* codon 61, 5′-AATTCCTTTTATTGAAACATCA-3′ for *KRAS* codon 146, 5′-TGATTTTGGTCTAGCTACA-3′ for *BRAF* codon 600, 5′-CCATAGAAAATCTTTCTCCT-3′ (RS1), 5′-TTCTCCTT/GCTT/CAGTGATTT-3′ (RS2), 5′-TAGAAAATCTTTCTCCTGCT-3′ (RS3) for *PIK3CA* exon 19, and 5′-GTTGTCCAGCCACCA-3′ for *PIK3CA* exon 20.

In addition, for a subset of 720 cases, tumor mutational profile was obtained from whole exome sequencing (WES), as previously described, for genes of interest (115 genes, Supplementary Table 3) without pyrosequencing data^43^. Briefly, DNA from tumor areas of tumor FFPE blocks were extracted along with paired normal DNA from tumor-free areas or resection margins and underwent hybrid capture with SureSelect v.2 Exome bait (Agilent Technologies) and sequencing with Illumina HiSeq 2000 instruments. Frequency of single nucleotide variants were stratified by MSI status and genes with significant mutations beyond background mutational level were considered for analysis. Genes with less than 5% non-silent mutation frequency in the dataset were excluded from the analysis (see Supplementary Table 1 for the full list of mutations included in the analysis).

### Quantitative detection of *Fusobacterium nucleatum* and *Bifidobacterium* genus in tumors

We performed a quantitative PCR assay to measure the amount of *Fusobacterium nucleatum* and *Bifidobacterium* genus DNA in the tumor tissue, as previous described^38,44^. The amount of *Fusobacterium nucleatum* and *Bifidobacterium* genus DNA in each tumor specimen were calculated as a relative value normalized to levels of human reference gene *SLCO2A1* using the 2^−ΔCt^ method^45^. Cases with any detectable *Bifidobacterium* DNA were categorized as low vs. high based on the median cut point amount of *Bifidobacterium*, while cases without detectable *Bifidobacterium* were categorized as negative. Due to a larger proportion of absence of *F. nucleatum* DNA in the samples, *F. nucleatum* was categorized as absent or present based on the detection of *F. nucleatum* DNA.

### Immunohistochemical analysis

We constructed tissue microarrays that included up to four cores from colorectal cancer and up to two cores from normal tissue blocks, as detailed in ref. ^46^. We use the standardized nomenclature system for proteins as recommended by the expert panel^47^.

Immunohistochemical analyses of PTGS2 (HGNC:9605; cyclooxygenase-2), nuclear CTNNB1 (HGNC:2514; beta-catenin), CD274 (HGNC:17635; PD-L1), PDCD1 (HGNC:8760; PD-1), and PDCD1LG2 (HGNC:18731; PD-L2) were performed using an anti-PTGS2 antibody (1:300 dilution; Cayman Chemical, Ann Arbor, MI, USA), anti-CTNNB1 antibody (1:400 dilution; BD Transduction Laboratories, Franklin Lakes, NJ, USA), anti-CD274 antibody (1:50 dilution; eBioscience, San Diego, CA), anti-PDCD1 antibody (1:1000 dilution; Clone EH33), and anti-PDCD1LG2 antibody (1:6000 dilution; clone 366C.9E5), respectively^46,48–50^. Anti-PDCD1 antibody and anti-PDCD1LG2 antibody were generated in the laboratory of G.J. Freeman at Dana-Farber Cancer Institute^51^.

### Multispectral immunofluorescence

Multispectral immunofluorescence, as previously described, was performed using deparaffinized 4 µm sections from tissue microarray blocks, and tissue microarray cores were sampled from different areas of tumor (i.e., center and periphery)^52^. Up to four tumor cores from each case were collected. Many cores also contain microscopic invasive edges (e.g., tumor budding), and features of those microscopic invasive edges were similar to those in the tumor periphery^53^. Primary antibodies against CD3 (1:75 dilution; clone F7.2.38; Dako; Agilent Technologies, Carpenteria, CA, USA), CD4 (1:50 dilution; clone 4B12; Dako), CD8 (1:150 dilution; clone C8/144B; Dako), CD45RO isoform of the PTPRC products (1:50 dilution; clone UCHL1; Dako), FOXP3 (1:100 dilution; clone 206D; Biolegend, San Diego, CA), and KRT (keratins, pan-cytokeratins) (combination of 1:40 dilution; clone AE1/AE3; Dako, and 1:400 dilution; clone C11; Cell signaling, Danvers, MA, USA), and DAPI (Catalog number FP1490, Akoya Biosciences, Marlborough, MA, USA) were detected using a tyramide signal amplification method and Opal fluorescent dyes (Akoya Biosciences). The stained slides were imaged using the multispectral imaging platform (Vectra 3.0, Akoya Biosciences) at ×200 magnification. Multispectral images of each core underwent first tissue segmentation to characterize regions of tumor epithelium and stroma based on KRT expression, using supervised machine-learning algorithms within Inform 2.4.1 (Akoya Biosciences). Following tissue segmentation, cell enumeration, and segmentation was performed using the DAPI signal to aid in identification of nuclei. Each cell was further segmented into nuclear, cytoplasmic and membranous compartments. A separate supervised machine-learning algorithm was used to identify T cells based upon a combination of cytomorphology and T-cell marker expression patterns. These single-cell data were then used to calculate T-cell subpopulation densities within separate regions. Aggregate tumor-level densities were then determined by calculating the average density (cells/mm^2^) for each subset across all regions from each patient.

### Statistical analysis

BART, an ensemble sum-of-trees model under a Bayesian paradigm, is an extension to the concepts of gradient boosting, whereby each tree $g\left({x;}{T}_{j}{M}_{j}\right)$ within an ensemble represents a portion of the final predicted outcome *Y*:

$$Y=\mathop{\sum }\limits_{j=1}^{m}g\left(x{\rm{;}}{T}_{j}{M}_{j}\right)+\varepsilon \, \qquad \varepsilon \sim N(0,{\sigma }^{2})$$

Under the Bayesian paradigm, a set of prior distributions is first determined for tree structure (*T*), the leaf parameters given the tree structure (*M|T*), and the error variance (*σ*^2^), as detailed in ref. ^11^. The prior distributions are then updated iteratively given the observed data by employing Markov Chain Monte Carlo (MCMC), which generates draws from the posterior distribution $P({T}_{1}^{M},\ldots ,{T}_{m}^{M},{\sigma }^{2}|y)$.

By setting a uniform prior on predictor variables as well as a prior that centers on shallow tree depths of 2–3 levels, the BART method enforces regularization with weak learners at each iteration. Through each iteration of MCMC using Gibbs sampling, the BART model grows, shrinks, or maintains tree structure by choosing variables, variable split points, and terminal contributions with respect to a probability distribution based on residual minimization. The posterior samples reflect the true underlying posterior probability distribution. Further summary statistics can then be performed to determine the expected values and credible intervals of parameters of interest.

Using data from 815 study participants (Fig. 1), we performed a random 80–20 training (*n* = 652) vs testing (*n* = 163) split for 5-year survival prediction. Overall, 75 variables were initially considered as predictors in the models. Supplementary Table 1 shows a full list of predictor variables used in this study.

Preprocessing was performed on all continuous variables. As T-cell densities in tumor were highly skewed, they were transformed using Yeo-Johnson transformation for normality^54^. Continuous variables and ordinal variables with more than two levels were then centered and scaled with mean of 0 and standard deviation of 1. BART, LASSO linear regression, GB, RF, adaptive boosting, support vector machine (SVM), and ANN algorithms were then performed on the training sets with parameters within a default tuning grid set by R *caret* package tuned by cross-validation, and the prediction performance on the validation sets was measured by ROC concordance statistics (area under ROC curve, AUC). To assess for internal stability of the predictors and model performance in terms of AUC, we performed a fivefold cross-validation with 80–20 training and validation split for each fold.

For primary analysis with BART models, all variables were considered; no imputation was performed, and missingness was included as a node-splitting option (see Fig. 1)^55^. For comparisons between learning algorithms, K-Nearest Neighbor imputation was performed on all variables prior to downstream analysis, as not all algorithms allow for missing data.

Important variables were determined via proportion of inclusion and permuted significance based on local procedure permutation methods across 1000 permutations^13^. In this exploratory analysis, variables were selected based on permuted significance at *P* value = 0.05 (level of suggestive evidence^56^) for ≥10 times across ten random runs (i.e., average of ≥1/5 folds of cross-validation). For important variables, partial dependence plots were generated by plotting outcome predictions against varying single predictor values, while holding all other variables constant in the trained model. Credible intervals were generated by obtaining the average and standard deviations of 1000 posterior samples of the BART model.

A BART risk prediction model was constructed using the selected variables, using leave-one-out training/testing split to estimate predicted survival probabilities for each patient with stage II or stage III colorectal cancer. Predicted survival probabilities were further categorized into equally sized risk quantiles (low risk, intermediate risk, and high risk) within all stage II–III patients. Survival analysis was conducted on the risk quantiles via Cox proportional hazards regression and log-rank test. Cox proportional hazards assumption was not satisfied, and therefore, hazards ratios (HRs) should be interpreted as weighted average HRs over time^57^. Multivariate Cox proportional hazards regression was performed with ordinal risk groups (low risk to high risk) and TNM stage, and ordinal risk groups with predictor variables of the BART risk model. Hazard ratios represent hazard ratios associated with one unit increase in each predictor variable unless otherwise coded as described above. Considering inherent multiple comparisons, we used the alpha level of 0.005 for significance with *P* value between 0.005 and 0.05 for suggestive evidence, as recommended by the expert statistical panel^56^. All *P* values represent two-sided testing. Risk prediction model calibration adequacy was assessed by Hosmer–Lemeshow goodness-of-fit test^58^.

All machine-learning algorithms were performed using the *Caret* package in R^59^, a wrapper API for specific machine-learning packages: *bartMachine*^60^*, randomForest, gbm, nnet, and e1071*. Partial dependence plots were generated using the *bartMachine* package in R. ROC plots were generated using the *pROC* package in R. Survival plots were generated using the *survminer* package in R. Cox proportional hazards models were generated using the *survival* package in R. Model calibration was analyzed via *plotCalibration* function in the *PredictABLE* package in R. Risk prediction model interface was designed using *Shiny* in R. All statistical analyses were performed using R 4.1.1.

### External validation with The Cancer Genome Atlas (TCGA) data

The most recent The Cancer Genome Atlas (TCGA) data (release date January 28, 2016) was extracted from the COADREAD (Colorectal Adenocarcinoma) project dataset using the R package *RTCGA*. Patients (*n* = 371) with stage II–III colorectal cancer and survival information were included in the validation set. Available variables, including positive and negative lymph node counts, depth of tumor invasion, age, tumor site, and microsatellite instability status, were pulled from the server and when necessary, reformatted to the same units as those reflected in the NHS/HPFS dataset. A single pathologist (M.Z.), blinded to other data, performed a pathological review of digital TCGA hematoxylin and eosin-stained tissue sections of all available cases and recorded the extent of extraglandular necrosis. As no colorectal cancer-specific survival information was available in TCGA, 5-year overall survival was used as a surrogate outcome. In survival analyses, censoring was set at 5 years because most colorectal cancer-specific deaths occur within 5 years of disease diagnosis, as observed in the NHS/HPFS cohorts.

### Reporting summary

Further information on research design is available in the Nature Research Reporting Summary linked to this article.

## Supplementary information

Supplementary information

REPORTING SUMMARY

## Data Availability

Due to participant confidentiality and privacy concerns, data are available upon reasonable written request. Further information including the procedures to obtain and access data from the Nurses’ Health Studies and Health Professionals Follow-up Study is described at https://www.nurseshealthstudy.org/researchers (contact email: nhsaccess@channing.harvard.edu) and https://sites.sph.harvard.edu/hpfs/for-collaborators/.

## Code availability

All code was implemented in R 4.1.1 using caret as the primary machine-learning package. All code and scripts to reproduce the experiments of this paper are available for noncommercial academic purposes upon reasonable written request. According to standard controlled access procedure, applications to use NHS/NHSII/HPFS resources will be reviewed by our External Collaborators Committee. An experimental version of the BART risk prediction model is publicly available for download at https://github.com/mm-zhao/BART.
