# Supplementary material for: Bayesian risk prediction model for colorectal cancer mortality through integration of clinicopathologic and genomic data
Source: NPJ Precis Oncol. 2023 Jun 10;7:57. doi: 10.1038/s41698-023-00406-8 (PMC10257677; doi:10.1038/s41698-023-00406-8)
Supplement: Supplementary file 1 — Supplementary information [file 41698_2023_406_MOESM1_ESM.pdf]

SUPPLEMENTARY INFORMATION

Supplementary Table 1. List of all seventy-five variables considered as predictors in the models.

Features (N = 75)				
Clinical (n = 11)	Pathological (n = 14)	Molecular (n = 33)	Immune (n = 15)	Bacterial (n = 2)
Gender,<br>Age (years),<br>Tumor site,<br>Family history,<br>Smoking history (pack-years)<br><br>**Pre-diagnosis factors:**<br>Body mass index,<br>Physical activity,<br>Alcohol intake,<br>Red meat intake (daily servings),<br>Regular aspirin use<br>Regular ibuprofen use	pT stage,<br>Positive LN count,<br>Negative LN count,<br>Tumor differentiation,<br>Extraglandular necrosis,<br>Signet ring cell component,<br>Extracellular mucinous component,<br>Lymphovascular invasion,<br>Perineural invasion<br><br>Immunohistochemical expression:<br>CD274 (PD-L1), CTNNB1 (beta-catenin), PDCD1 (PD-1), PDCD1LG2 (PD-L2), PTGS2 (cyclooxygenase 2)	MSI,<br>CIMP,<br>LINE-1 methylation level,<br>Neoantigen load<br><br>**Pyrosequencing (mutations):**<br>*KRAS,*<br>*BRAF,*<br>*PIK3CA*<br><br>**Genes with non-silent mutations in > 5% of patients included in WES:**<br>*ACVR2A, ADAMTS3, APC, ARID1A, ATXN2L, AXIN2, B2M, BCL9L, BMPR2, CHD4, FBXW7, FHOD3, FRMD4A, HLA-B, LARP4B, MUC17, PTEN, RNF43, SMAD2, SMAD4, SOX9, TCF7L2, TCF20, TP53, UBR1, ZFP36L2*	Lymphocytic reaction score,<br>TIL,<br>Intratumoral periglandular reaction,<br>Peritumoral reaction,<br>Crohn's-like reaction<br><br>**Tumor & stroma T cell densities:** Regulatory T cells,<br>Memory helper T cells,<br>Naïve helper T cells,<br>Memory cytotoxic T cells,<br>Naïve cytotoxic T cells	*Fusobacterium nucleatum* DNA,<br>*Bifidobacterium spp.* DNA

Abbreviations: CIMP, CpG island methylator phenotype; LINE-1, long interspersed nucleotide element-1; LN, lymph node; MSI, microsatellite instability; TIL, tumor-infiltrating lymphocytes; WES, whole exome sequencing.

Supplementary Table 2. BART model performance for five-year colorectal cancer-specific survival across 5-fold cross validation, using overall stage, pT stage, or pN stage alone as predictor variable vs 7 significant variables. Median values are bolded.

	C-Statistics			
Folds	Stage	pT stage	pN stage	7 variables
1	0.65	0.41	0.63	0.77
2	0.55	0.50	0.55	0.67
3	**0.62**	**0.47**	**0.58**	0.83
4	0.49	0.47	0.54	0.73
5	0.66	0.46	0.71	**0.74**

Supplementary Table 3: Full list of 115 genes with single nucleotide variations in whole exome sequencing data on colorectal cancer in the Health Professionals Follow-up Study and the Nurses' Health Study.

ABCF2, ACOXL, ACVR2A, ADAM30, ADAMTS3, ADD2, AHI1, APC, ARHGAP5, ARID1A, ARPC1B, ASXL1, ATP6V1B1, ATXN2L, AXIN2, B2M, BCL9L, BMPR2, C6orf136, C7orf31, CASD1, CASP8, CHD4, CTCF, CTNNB1, CUL5, DAO, DGKA, DIAPH1, DRD3, DUSP16, EI24, ELF3, FAM171B, FBXW7, FHOD3, FLYWCH1, FRMD4A, GDF5, GORASP1, GRHPR, HEATR2, HLA-A, HLA-B, HSPA1L, HTR3C, IL7R, ING1, ITIH1, KLF3, KLF5, LARP4B, LIMK1, MAP2K1, MAP2K7, MARK2, MGAT3, MOV10, MST4, MUC17, MVK, MYBL2, NAT10, NBN, NCAPD3, NEK2, NKTR, NRAS, OCRL, PABPC1L, PACSIN1, PAN3, PAX6, PCBP1, PLEKHA6, PRKCQ, PTEN, RANBP9, RB1, RBM10, RBM12, RNF128, RNF43, RUFY1, SAMM50, SERPING1, SIN3A, SMAD2, SMAD4, SNAPC1, SOAT1, SOX9, SRRT, SSH1, SYNCRIP, SYNGR2, TCF20, TCF7, TCF7L2, TDRD1, TEX14, TGIF1, TMEM201, TNFRSF4, TNFRSF9, TP53, TPTE2, UBR1, USP5, WDR86, WNT16, XYLT2, ZBTB20, ZFP36L2, ZNRF3

Supplementary Figure 1.

Survival based on BART risk model prediction quantiles and stage

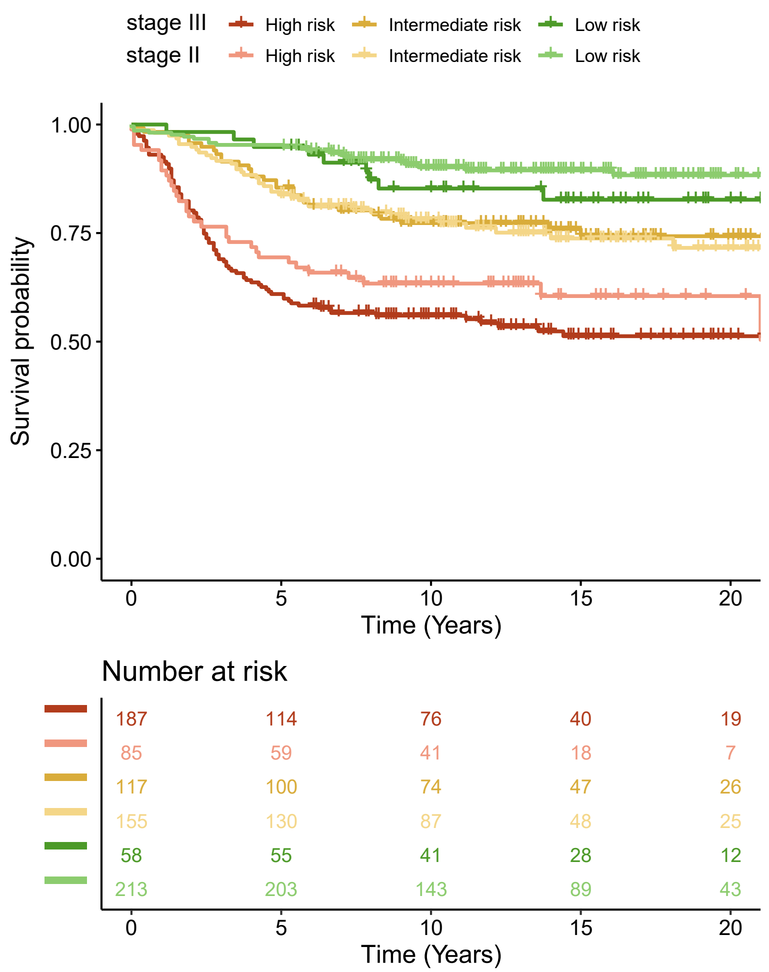

Ref: high risk stage III	HR	95% CI	P-value
High risk stage II	0.79	0.53-1.19	0.26
Int risk stage III	0.43	0.28-0.65	<0.0001
Int risk stage II	0.42	0.29-0.62	<0.0001
Low risk stage III	0.24	0.12-0.48	<0.0001
Low risk stage II	0.16	0.10-0.26	<0.0001
**Overall**			<0.0001

Kaplan-Meier plot for survival in patients with Stage II/III colorectal cancer in TCGA dataset. Table shows Cox proportional hazards model using risk quantiles and overall P-value by Log-rank test.

Abbreviations: BART, Bayesian additive regression trees; CI, confidence interval; HR, hazard ratio.

Supplementary Figure 2.

ROC curve for TCGA external validation

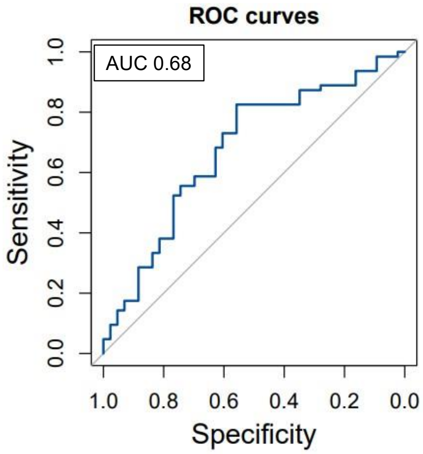

ROC curve for external validation of BART risk model with TCGA data

Abbreviations: AUC, area under the ROC curve; ROC, Receiver Operating Characteristics.

Supplementary Figure 3.

User interface for BART risk prediction model

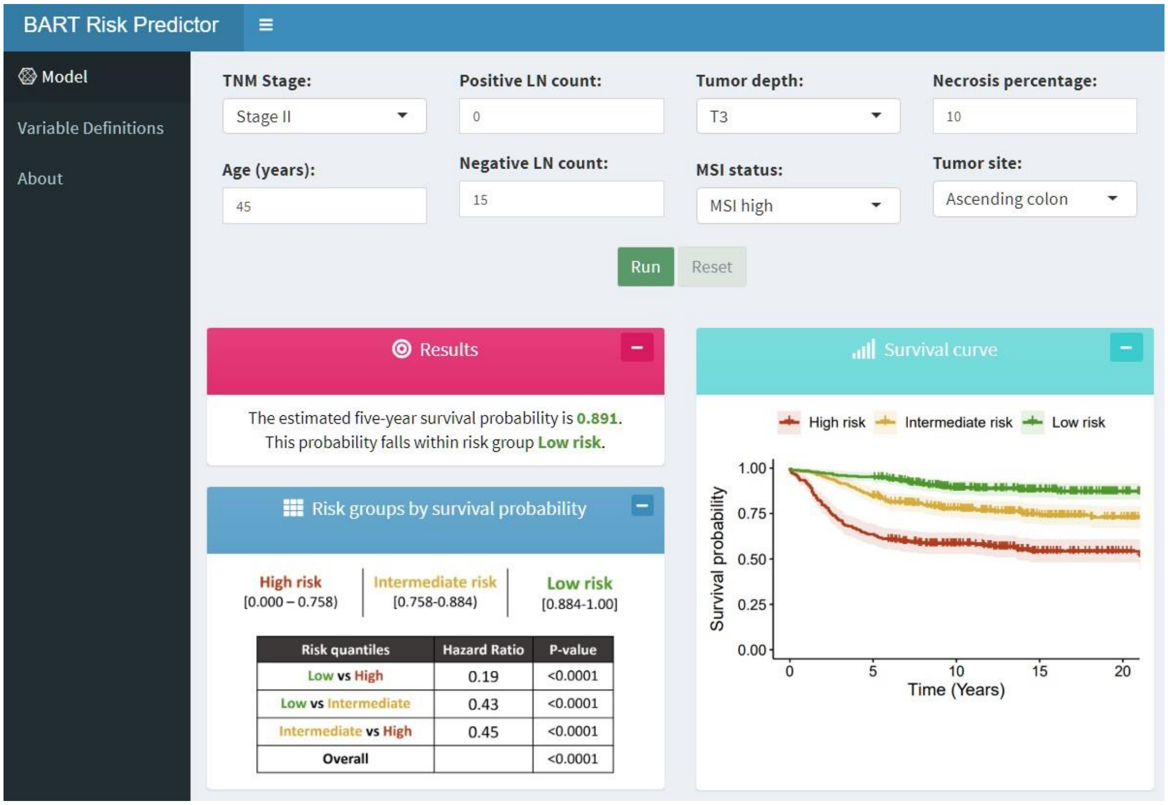

User interface takes 7 variables as input, runs the BART risk prediction model, and outputs survival probability along with risk category (high, intermediate, and low risk). An experimental version is available for download at https://github.com/mm-zhao/BART.
