# Supplementary material for: Bayesian risk prediction model for colorectal cancer mortality through integration of clinicopathologic and genomic data
Source: NPJ Precis Oncol. 2023 Jun 10;7:57. doi: 10.1038/s41698-023-00406-8 (PMC10257677; doi:10.1038/s41698-023-00406-8)
Supplement: Supplementary file 2 — REPORTING SUMMARY [file 41698_2023_406_MOESM2_ESM.pdf]

nature portfolio

Corresponding author(s): Shuji Ogino, Melissa Zhao

Last updated by author(s): Apr 8, 2023

Reporting Summary

Nature Portfolio wishes to improve the reproducibility of the work that we publish. This form provides structure for consistency and transparency in reporting. For further information on Nature Portfolio policies, see our Editorial Policies and the Editorial Policy Checklist.

Statistics

For all statistical analyses, confirm that the following items are present in the figure legend, table legend, main text, or Methods section.

n/a	Confirmed	
☐	☒	The exact sample size (*n*) for each experimental group/condition, given as a discrete number and unit of measurement
☐	☒	A statement on whether measurements were taken from distinct samples or whether the same sample was measured repeatedly
☐	☒	The statistical test(s) used AND whether they are one- or two-sided<br>*Only common tests should be described solely by name; describe more complex techniques in the Methods section.*
☐	☒	A description of all covariates tested
☐	☒	A description of any assumptions or corrections, such as tests of normality and adjustment for multiple comparisons
☐	☒	A full description of the statistical parameters including central tendency (e.g. means) or other basic estimates (e.g. regression coefficient) AND variation (e.g. standard deviation) or associated estimates of uncertainty (e.g. confidence intervals)
☐	☒	For null hypothesis testing, the test statistic (e.g. *F*, *t*, *r*) with confidence intervals, effect sizes, degrees of freedom and *P* value noted<br>*Give P values as exact values whenever suitable.*
☐	☒	For Bayesian analysis, information on the choice of priors and Markov chain Monte Carlo settings
☐	☒	For hierarchical and complex designs, identification of the appropriate level for tests and full reporting of outcomes
☐	☒	Estimates of effect sizes (e.g. Cohen's *d*, Pearson's *r*), indicating how they were calculated

Our web collection on statistics for biologists contains articles on many of the points above.

Software and code

Policy information about availability of computer code

Data collection	No software used for data collection.
Data analysis	All machine learning algorithms were performed using the Caret package in R,38 a wrapper API for specific machine learning packages: bartMachine, randomForest, gbm, nnet, and e1071. Partial dependence plots were generated using the bartMachine package in R. ROC plots were generated using the pROC package in R. Survival plots were generated using the survminer package in R. Cox proportional hazards models were generated using the survival package in R. Model calibration was analyzed via plotCalibration function in the PredictABLE package in R. Risk prediction model interface was designed using Shiny in R. All statistical analyses were performed using R 4.1.1.

For manuscripts utilizing custom algorithms or software that are central to the research but not yet described in published literature, software must be made available to editors and reviewers. We strongly encourage code deposition in a community repository (e.g. GitHub). See the Nature Portfolio guidelines for submitting code & software for further information.

Data

Policy information about availability of data

All manuscripts must include a data availability statement. This statement should provide the following information, where applicable:

- Accession codes, unique identifiers, or web links for publicly available datasets
- A description of any restrictions on data availability
- For clinical datasets or third party data, please ensure that the statement adheres to our policy

Due to participant confidentiality and privacy concerns, data are available upon reasonable written request. Further information including the procedures to obtain and access data from the Nurses' Health Studies and Health Professionals Follow-up Study is described at https://www.nurseshealthstudy.org/researchers (contact email: nhsaccess@channing.harvard.edu) and https://sites.sph.harvard.edu/hpfs/for-collaborators/.

Research involving human participants, their data, or biological material

Policy information about studies with human participants or human data. See also policy information about sex, gender (identity/presentation), and sexual orientation and race, ethnicity and racism.

Reporting on sex and gender	Sex was a variable examined in this study, and data collected from both sexes are reported in the study, from Nurses' Health Study and Health Professionals Follow-up Study.
Reporting on race, ethnicity, or other socially relevant groupings	These variables were not used in the study. The study includes a prospective nation-wide cohort of participants and information on race, ethnicity, and other social variables are not examined in this study.
Population characteristics	The population studied is patients with stage II/III colorectal cancer with tissue-level information available.
Recruitment	For the Nurses' Health Study, married registered nurses, aged 30 to 55 in 1976, who lived in the 11 most populous states, and whose nursing boards agreed to supply NHS with their members' names and addresses, were eligible to be enrolled in the cohort if they responded to the NHS baseline questionnaire. The NHS cohort was then established by a series of three mailings of the baseline questionnaire. The first mailing to all 238,026 nurses occurred in June 1976, with the final mailing to non-respondents in December 1976. Overall, 121,700 women returned a completed questionnaire. After excluding 65,613 questionnaires that could not be delivered, the response rate was approximately 71% (121,700 of 172,413).<br><br>A similar method was employed to establish the Health Professionals Follow-Up Study cohort. 51,529 men in health professions were recruited to participate in the study. This group is composed of 29,683 dentists, 4,185 pharmacists, 3,745 optometrists, 2,220 osteopath physicians, 1,600 podiatrists, and 10,098 veterinarians.
Ethics oversight	The study protocol was approved by the institutional review boards of the Brigham and Women's Hospital and Harvard T.H. Chan School of Public Health (Boston, MA, USA), and those of participating registries as required.

Note that full information on the approval of the study protocol must also be provided in the manuscript.

Field-specific reporting

Please select the one below that is the best fit for your research. If you are not sure, read the appropriate sections before making your selection.

☒ Life sciences ☐ Behavioural & social sciences ☐ Ecological, evolutionary & environmental sciences

For a reference copy of the document with all sections, see nature.com/documents/nr-reporting-summary-flat.pdf

Life sciences study design

All studies must disclose on these points even when the disclosure is negative.

Sample size	Sample size is based on all patients in the NHS/HPFS cohorts with stage II/III colorectal cancer and tissue samples available at time of diagnosis
Data exclusions	No data were excluded from analysis, if they satisfied the inclusion criteria
Replication	Models were run multiple times and code went through the code checking process at the overseeing institution to ensure reproducibility.
Randomization	Training and validation sets are randomly split in the dataset prior to model learning.
Blinding	Investigators analyzing the data were blinded to all variables.

Reporting for specific materials, systems and methods

We require information from authors about some types of materials, experimental systems and methods used in many studies. Here, indicate whether each material, system or method listed is relevant to your study. If you are not sure if a list item applies to your research, read the appropriate section before selecting a response.

Materials & experimental systems

n/a	Involved in the study
☐	☒ Antibodies
☒	☐ Eukaryotic cell lines
☒	☐ Palaeontology and archaeology
☒	☐ Animals and other organisms
☒	☐ Clinical data
☒	☐ Dual use research of concern
☒	☐ Plants

Methods

n/a	Involved in the study
☒	☐ ChIP-seq
☒	☐ Flow cytometry
☒	☐ MRI-based neuroimaging

Antibodies

Antibodies used

Immunohistochemical analyses of PTGS2 (HGNC:9605; cyclooxygenase-2), nuclear CTNNB1 (HGNC:2514; beta-catenin), CD274 (HGNC:17635; PD-L1), PDCD1 (HGNC:8760; PD-1), and PDCD1LG2 (HGNC:18731; PD-L2) were performed using an anti-PTGS2 antibody (dilution, 1:300; Cayman Chemical, Ann Arbor, MI, USA), anti-CTNNB1 antibody (dilution, 1:400; BD Transduction Laboratories, Franklin Lakes, NJ, USA), anti-CD274 antibody (1:50 dilution; eBioscience, San Diego, CA), anti-PDCD1 antibody (1:1000 dilution; Clone EH33), and anti-PDCD1LG2 antibody (1:6,000 dilution; clone 366C.9E5), respectively. Anti-PDCD1 antibody and anti-PDCD1LG2 antibody were generated in the laboratory of G.J. Freeman at Dana-Farber Cancer Institute.

Validation

Validation of antibody use are detailed in the following references cited in the paper:
Chan AT, Ogino S, Fuchs CS. Aspirin and the risk of colorectal cancer in relation to the expression of COX-2. N Engl J Med. 2007;356(21):2131-2142. doi:10.1056/NEJMoa067208
Masugi Y, Nishihara R, Yang J, et al. Tumour CD274 (PD-L1) expression and T cells in colorectal cancer. Gut. 2017;66(8):1463-1473. doi:10.1136/gutjnl-2016-311421
Morikawa T, Kuchiba A, Yamauchi M, et al. Association of CTNNB1 (beta-catenin) alterations, body mass index, and physical activity with survival in patients with colorectal cancer. JAMA. 2011;305(16):1685-1694. doi:10.1001/jama.2011.513
Masugi Y, Nishihara R, Hamada T, et al. Tumor PDCD1LG2 (PD-L2) Expression and the Lymphocytic Reaction to Colorectal Cancer. Cancer Immunol Res. 2017;5(11):1046-1055. doi:10.1158/2326-6066.CIR-17-0122
Ansell SM, Lesokhin AM, Borrello I, et al. PD-1 blockade with nivolumab in relapsed or refractory Hodgkin's lymphoma. N Engl J Med. 2015;372(4):311-319. doi:10.1056/NEJMoa1411087
